# Induction chemotherapy in the treatment of nasopharyngeal carcinoma: Clinical outcomes and patterns of care

**DOI:** 10.1002/cam4.1626

**Published:** 2018-07-14

**Authors:** Prashant Gabani, Justin Barnes, Alexander J. Lin, Soumon Rudra, Peter Oppelt, Douglas Adkins, Jason T. Rich, Jose P. Zevallos, Mackenzie D. Daly, Hiram A. Gay, Wade L. Thorstad

**Affiliations:** ^1^ Department of Radiation Oncology Washington University School of Medicine Saint Louis MO USA; ^2^ Saint Louis University School of Medicine Saint Louis MO USA; ^3^ Division of Medical Oncology Washington University School of Medicine Saint Louis MO USA; ^4^ Department of Otolaryngology Washington University School of Medicine Saint Louis MO USA

**Keywords:** Induction Chemotherapy, Nasopharyngeal Carcinoma, Nasopharynx, NCDB, Propensity Score Matching

## Abstract

The role of induction chemotherapy in nasopharyngeal carcinoma (NPC) remains controversial. The primary aim of this study was to use the National Cancer Database to evaluate the patterns of care of induction chemotherapy in NPC and its impact on overall survival (OS). Patients with NPC from 2004 to 2014 were obtained from the NCDB. Patients were considered to have received induction chemotherapy if it was started ≥43 days before the start of RT and concurrent CRT if chemotherapy started within 21 days after the start of RT. Propensity score matching was used to control for selection bias. Cox proportional hazards model was used to determine significant predictors of OS. Logistic regression model was used to determine predictors of the use of induction chemotherapy. Significance was defined as a *P* value <.05. A total of 4857 patients were identified: 4041 patients (87.2%) received concurrent CRT and 816 patients (16.8%) received induction chemotherapy. The use of induction therapy remained stable between 2004 and 2014. Younger patients and those with higher T‐ and N‐stage had a higher likelihood of being treated with induction chemotherapy. The 5‐year OS in patients treated with induction chemotherapy and CRT was 66.3% vs 69.1%, respectively (*P* = .25). There was no difference in OS when these two groups were analyzed after propensity score matching. No differences in OS existed between these treatment groups in patients with T3‐T4N1 or TanyN2‐3 disease (*P* = .76). Propensity score matching also did not reveal any difference in OS in patients with T3‐T4N1 or TanyN2‐3 disease. The use of induction chemotherapy has remained stable in the last decade. In this study of patients with NPC, induction chemotherapy was not associated with improved OS compared to CRT alone.

## 1 INTRODUCTION

Nasopharyngeal carcinoma (NPC) accounts for approximately 3000 new cases in the United States each year and approximately 80 000 new cases worldwide, with the majority of them diagnosed in Southeast and Eastern Asia, and Northern Africa.^1^ Given its anatomical location and the proximity to critical organs, radiation therapy (RT) is the primary local modality in contrast to other head and neck malignancies where surgery may still play a major role. As such, prior to the advent of megavoltage RT, NPC had a poor prognosis; however, modern RT technology, especially Intensity Modulated RT, and concurrent chemotherapy has resulted in >70% 5‐year overall survival.^2^, ^3^, ^4^, ^5^

Several studies in the early 1990s and 2000s demonstrated that concurrent chemoradiation (CRT) followed by adjuvant cisplatin improved survival compared to RT alone and established this as the standard of care for newly diagnosed NPC.^2^, ^5^ Furthermore, the use of 3‐dimensional imaging (CT and MRI) along with improvement in RT technology has resulted in excellent local control rates.^6^ However, distant failure still remains the main source of mortality in this patient population. There is a notion that additional chemotherapy, either in the form of induction therapy or adjuvant therapy, may improve distant control, especially in patients with locally advanced disease, resulting in an improved overall survival.

There have been several studies that have evaluated additional cycles of chemotherapy in the adjuvant setting without finding significant survival benefit.^5^, ^7^ The primary limitation of the preceding trials has been a low compliance rate of adjuvant chemotherapy with approximately 40‐50% patients not completing the prescribed chemotherapy regimen.^7^ Alternatively, induction chemotherapy is thought to be better tolerated and may result in early eradication of micrometastasis, leading to the hypothesis that it may result in improved survival. However, this has been controversial as several studies have found a survival benefit with this approach, while others have not.^8^, ^9^, ^10^ A meta‐analysis based on individual patient data has showed no benefit with induction chemotherapy.^11^ In this study, we aim to use the National Cancer Database (NCDB) to study the patterns of care in the use of induction chemotherapy and evaluate the survival benefit of induction chemotherapy in NPC.

## 2 MATERIALS AND METHODS

The NCDB is a joint project of the American Cancer Society and the American College of Surgeons Commission on Cancer. The American College of Surgeons has executed a Business Associate Agreement that includes a data use agreement with each of its Commission on Cancer accredited hospitals. The NCDB, established in 1989, is a nationwide, facility‐based, comprehensive clinical surveillance resource oncology data set that currently captures 70% of all newly diagnosed malignancies in the US annually. Data elements are collected and submitted to the NCDB from commission‐accredited oncology registries using standardized coding and data item definitions, including details not available from the Surveillance, Epidemiology, and End Results (SEER) registry, such as RT dose/technique, chemotherapy use/timing, and comorbidities.^12^

**Figure 1:**
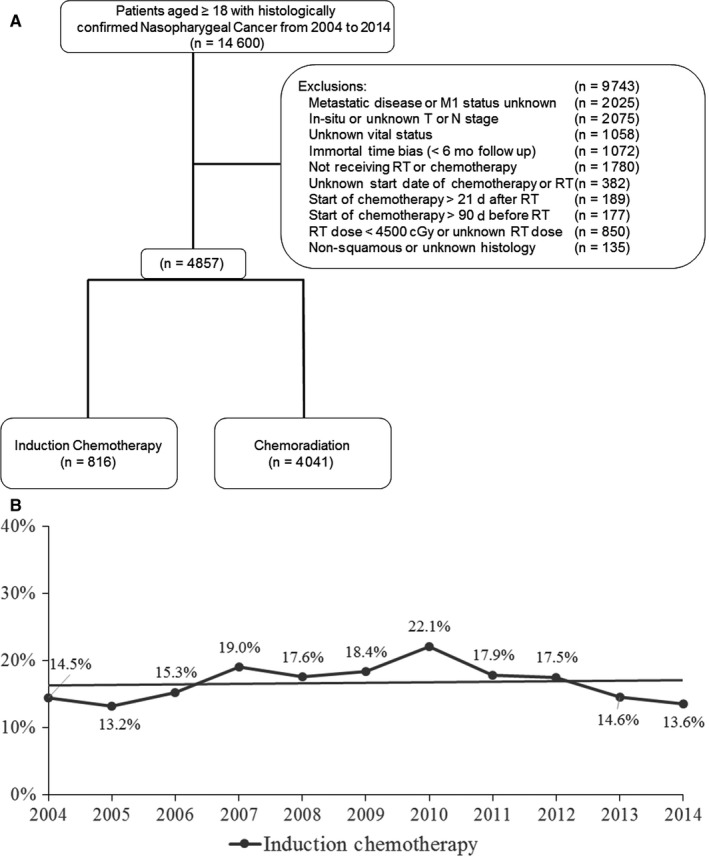
A, CONSORT diagram. B, Trends in the utilization of induction chemotherapy in nasopharyngeal carcinoma

De‐identified data for patients with newly diagnosed NPC from 2004 to 2014 were obtained from the NCDB participant user file. Inclusion and exclusion criteria are summarized in the CONSORT diagram (Figure 1A). Of the 14 600 patients identified, 4857 were included in the final analysis. Patients were considered to have received induction chemotherapy if it was started ≥43 days before the start of RT. This time point was chosen as it would allow for administration of 2‐3 cycles of induction chemotherapy used in recent trials.^7^, ^8^, ^10^ Patients were considered to have received concurrent CRT if chemotherapy started within 21 days after the start of RT as this would allow for overlap of at least 2‐3 cycles of concurrent chemotherapy with RT. Patients not meeting this criteria for induction and concurrent chemotherapy were excluded. It is also important to mention here that the use of adjuvant chemotherapy after completion of concurrent CRT could not be determined in this study due to the limitations of the database. To account for the immortal time bias, patients with <6 months of follow‐up or those who died <6 months after diagnosis were excluded. The 6 month time frame was chosen as it would allow all definitive treatment to be completed.

Given that a definitive RT dose for NPC in most recent trials is 70 Gy,^8^, ^13^ we categorized patients as those receiving <70 Gy or ≥70 Gy. For the purpose of this categorization, patients receiving 69.96 Gy were considered to have received 70 Gy since 69.96 Gy is also considered to be a definitive RT dose for NPC. Of note, multiple RT dose levels including 69, 68, 67, and 66 Gy were also analyzed and the results of this study remained unchanged (Figure S1). Other variables were categorized as previously published by Seisen et al^14^. Insurance and education variables were categorized into high and low, referring to the highest two quartiles and the lowest two quartiles, respectively.

Categorical data were summarized by frequency counts and percentages. Continuous variables were compared using the Wilcoxon test, and categorical variables were compared using Fisher's exact test. The primary end point was overall survival (OS), which was defined as the time from the date of their diagnosis to the date of death. OS rates were determined using the Kaplan‐Meier method and were compared between groups using log‐rank statistics. The Cox proportional hazards model was used to determine significant predictors of OS and to estimate hazard ratios (HRs) as well as associated 95% confidence intervals (CI). Logistic regression model was used to determine significant predictors of the use of induction chemotherapy and to estimate odds ratio (OR) as well as the associated 95% CI. Variables were included in the multivariable analysis only if significant on univariable analysis.

To reduce selection bias in this study, methods used by Seisen et al^14^ in their recent NCDB publication were utilized. Briefly, to account for selection bias, observed differences in baseline characteristics between the complete cases of the two groups were controlled for with inverse probability of treatment weighting (IPTW)‐adjusted analysis. The goodness‐of‐fit statistic of the propensity score model, including linear or nonlinear covariates categorized with clinically relevant cutoffs, was assessed using the method described by Lemeshow and Hosmer.^15^ Covariate balance was evaluated using the standardized difference approach and Kernel density plots (Figure S2). Statistical analyses were performed using SPSS, Version 23, and R, Version 3.3.2. Significance was defined as a *P* value <.05. All statistical tests were two sided.

## 3 RESULTS

A total of 4857 patients were included in the analysis based on the previously mentioned selection criteria: 4041 patients (87.2%) received concurrent CRT and 816 patients (16.8%) received induction chemotherapy (Table 1). There were several differences in patient characteristics that were identified between the two groups. Patients who received induction therapy were identified to be younger, had higher T‐stage and N‐stage, and received <70 Gy RT dose. More patients in the CRT alone group were treated with ≥70 Gy (60.2% vs 54.4%, *P* = .01).

**Table 1:** Baseline characteristics

	ChemoRT	Induction Chemotherapy	*P*‐value
	n = 4041 (%)	n = 816 (%)	
Age			
Mean (SD)	53.9 (13.3)	51.1 (13.6)	<.001
Sex			
Male	2842 (70.3)	619 (75.9)	.007
Female	1199 (29.7)	197 (24.1)	
Year of diagnosis			
2004	259 (6.4)	44 (5.4)	.007
2005	308 (7.6)	47 (5.8)	
2006	299 (7.4)	54 (6.6)	
2007	332 (8.2)	78 (9.6)	
2008	326 (8.1)	70 (8.6)	
2009	360 (8.9)	81 (9.9)	
2010	394 (9.8)	112 (13.7)	
2011	400 (9.9)	87 (10.7)	
2012	410 (10.1)	87 (10.7)	
2013	469 (11.6)	80 (9.8)	
2014	484 (12.0)	76 (9.3)	
Charlson/Deyo score			
0	3488 (86.3)	709 (86.9)	.57
1	448 (11.1)	91 (11.2)	
2	105 (2.6)	16 (2.0)	
Race			
White	2505 (62.7)	514 (63.5)	.008
African American	534 (13.4)	134 (16.6)	
Other	956 (23.9)	161 (19.9)	
Facility type			
Academic	1528 (43.7)	287 (43.0)	.73
Non Academic	1969 (56.3)	381 (57.0)	
Facility location			
East	1568 (44.8)	298 (44.6)	.06
Central	1277 (36.5)	268 (40.1)	
West	652 (18.6)	102 (15.3)	
Insurance status			
Private	2253 (56.6)	425 (53.2)	<.001
Medicaid or other Govt	600 (15.1)	165 (20.7)	
Medicare	889 (22.3)	136 (17.0)	
Uninsured	240 (6.0)	73 (9.1)	
Education level			
High	2108 (52.6)	381 (47.0)	.004
Low	1903 (47.4)	430 (53.0)	
Income level			
High	2326 (58.0)	454 (56.0)	.29
Low	1683 (42.0)	357 (44.0)	
County type			
Metro	3381 (85.9)	668 (83.9)	.07
Urban	506 (12.9)	111 (13.9)	
Rural	47 (1.2)	17 (2.1)	
Clinical T stage			
T1	1084 (26.8)	176 (21.6)	<.001
T2	1192 (29.5)	191 (23.4)	
T3	774 (19.2)	159 (19.5)	
T4	991 (24.5)	290 (35.5)	
Clinical N stage			
N0	844 (20.9)	150 (18.4)	<.001
N1	1281 (31.7)	219 (26.8)	
N2	1591 (39.4)	332 (40.7)	
N3	325 (8.0)	115 (14.1)	
RT dose			
≥7000 cGy	2447 (60.6)	441 (54.0)	.001
<7000 cGy	1594 (39.4)	375 (46.0)	

The utilization of induction chemotherapy has remained stable between 2004 and 2014, ranging between 13.2% and 22.1% (Figure 1B). Correlates for the receipt of induction chemotherapy were determined using univariable and multivariable modeling (Table 2). On univariable analysis, it was found that patients who were younger, had Medicaid or were uninsured, had lower education, and had T4 or N3 disease had a higher likelihood of receiving induction chemotherapy. Additionally, patients who received induction chemotherapy had a higher likelihood of being treated with <70 Gy. On multivariable analysis, patients with lower education (OR 1.35, 95% CI 1.08‐1.68, *P* = .01), T4 disease (OR 1.90, 95% CI 1.46‐2.48, *P* < .001), N3 disease (OR 2.47, 95% CI 1.72‐3.56, *P* < .001), and patients receiving <70 Gy RT (OR 1.28, 95% CI 1.05‐1.55, *P* = .02) had a higher likelihood of being treated with induction therapy. Conversely, older patients (OR 0.98, 95% CI 0.97‐0.99, *P* < .001) had a lower likelihood of being treated with induction therapy (Table 3).

**Table 2:** Univariable and multivariable analysis on factors predictive for receipt of induction chemotherapy

	Univariable analysis		Multivariable analysis	
	OR (95% CI)	*P*‐value	OR (95% CI)	*P*‐value
Age				
Continuous	0.98 (0.98‐0.99)	<.001	0.98 (0.97‐0.99)	<.001
Sex				
Male	Ref		Ref	
Female	0.75 (0.63‐ 0.90)	.001	NS	
Year of diagnosis				
2004	Ref		Ref	
2005	0.90 (0.56‐1.40)	.64	NS	
2006	1.06 (0.69‐1.64)	.78	NS	
2007	1.38 (0.92‐2.07)	.12	NS	
2008	1.26 (0.84‐1.91)	.26	NS	
2009	1.32 (0.89‐1.98)	.17	NS	
2010	1.67 (1.14‐2.45)	.008	NS	
2011	1.28 (0.86‐1.90)	.22	NS	
2012	1.25 (0.84‐1.85)	.27	NS	
2013	1.00 (0.67‐1.50)	.98	NS	
2014	0.92 (0.62‐1.38)	.70	NS	
Charlson index				
0	Ref		Ref	
1	1.00 (0.79‐1.27)	.99	NS	
2	0.75 (0.44‐1.28)	.29	NS	
Race				
White	Ref		Ref	
African American	1.22 (0.99‐1.51)	.06	NS	
Other	0.81 (0.68‐0.99)	.04	NS	
Facility type				
Academic	Ref		Ref	
Non Academic	1.03 (0.87‐1.22)	.73	NS	
Facility location				
East	Ref		Ref	
Central	1.10 (0.92‐1.32)	.28	NS	
West	0.82 (0.65‐1.05)	.12	NS	
Insurance status				
Private	Ref		Ref	
Medicaid or Other Govt	1.46 (1.19‐1.78)	<.001	NS	
Medicare	0.81 (0.66‐1.00)	.05	NS	
Uninsured	1.61 (1.22‐2.14)	.001	NS	
Education level				
High	Ref		Ref	
Low	1.25 (1.08‐1.45)	.004	1.35 (1.08‐1.68)	0.01
Income level				
High	Ref		Ref	
Low	1.09 (0.93‐1.26)	.28	NS	
County type				
Metro	Ref		Ref	
Urban	1.11 (0.89‐1.39)	.36	NS	
Rural	1.83 (.04‐3.21)	.04	NS	
Clinical T stage				
T1	Ref		Ref	
T2	0.99 (0.79‐1.23)	.91	NS	
T3	1.26 (1.00‐1.60)	.05	NS	
T4	1.80 (1.47‐ 2.22)	<.001	1.90 (1.46‐2.48)	<.001
Clinical N stage				
N0	Ref		Ref	
N1	0.96 (0.77‐1.20)	.74	NS	
N2	1.17 (0.95‐1.45)	.12	NS	
N3	1.99 (1.51‐2.62)	<.001	2.47 (1.72‐3.56)	<.001
RT Dose				
≥7000 cGy	Ref		Ref	
<7000 cGy	1.31 (1.12‐1.52)	.001	1.28 (1.05‐1.55)	.02

**Table 3:** Univariable and multivariable analysis for factors predictive of overall survival

	Univariable analysis		Multivariable analysis	
	HR (95% CI)	*P*‐value	HR (95% CI)	*P*‐value
Age				
Continuous	1.04 (1.03‐1.04)	<.001	1.03 (1.02‐1.04)	<.001
Sex				
Male	Ref		Ref	
Female	0.94 (0.84‐1.05)	.30	NS	
Year of diagnosis				
2004	Ref		Ref	
2005	1.23 (0.98‐1.55)	.08	NS	
2006	1.29 (1.02‐1.62)	.03	NS	
2007	1.14 (0.90‐1.44)	.28	NS	
2008	1.25 (0.99‐1.58)	.06	NS	
2009	1.11 (0.88‐1.42)	.38	NS	
2010	1.12 (0.88‐1.42)	.36	NS	
2011	1.15 (0.90‐1.47)	.28	NS	
2012	0.98 (0.75‐1.27)	.87	NS	
2013	1.10 (0.85‐1.44)	.46	NS	
2014	0.97 (0.72‐1.30)	.82	NS	
Charlson index				
0	Ref		Ref	
1	1.62 (1.40‐1.86)	<.001	1.26 (1.08‐1.46)	.003
2	2.23 (1.73‐2.88)	<.001	1.52 (1.16‐1.98)	.002
Race				
White	Ref		Ref	
African American	0.90 (0.78‐1.04)	.16	NS	
Other	0.54 (0.47‐0.62)	<.001	0.61 (0.52‐0.72)	<.001
Facility type				
Academic	Ref		Ref	
Non Academic	1.07 (0.96‐1.19)	.21	NS	
Facility location				
East	Ref		Ref	
Central	1.09 (0.97‐1.22)	.16	NS	
West	1.01 (0.88‐1.17)	.85	NS	
Insurance status				
Private	Ref		Ref	
Medicaid or Other Govt	1.66 (1.44‐1.92)	<.001	1.59 (1.35‐1.87)	<.001
Medicare	2.69 (2.39‐3.02)	<.001	1.52 (1.30‐1.78)	<.001
Uninsured	1.71 (1.40‐2.10)	<.001	1.62 (1.29‐2.03)	<.001
Education level				
High	Ref		Ref	
Low	1.30 (1.17‐1.43)	<.001	NS	
Income level				
High	Ref		Ref	
Low	1.39 (1.26‐1.54)	<.001	NS	
County type				
Metro	Ref		Ref	
Urban	1.34 (1.16‐1.53)	<.001	NS	
Rural	1.21 (0.81‐1.82)	.35	NS	
Clinical T stage				
T1	Ref		Ref	
T2	1.35 (1.17‐1.57)	<.001	1.29 (1.10‐1.52)	.002
T3	1.62 (1.38‐1.90)	<.001	1.62 (1.37‐1.93)	<.001
T4	2.19 (1.90‐2.52)	<.001	2.32 (1.98‐2.71)	<.001
Clinical N stage				
N0	Ref		Ref	
N1	0.71 (0.62‐0.82)	<.001	NS	
N2	0.86 (0.76‐0.98)	.02	NS	
N3	1.40 (1.17‐1.67)	<.001	1.84 (1.51‐2.24)	<.001
RT dose				
≥7000 cGy	Ref		Ref	
<7000	1.16 (1.05‐1.28)	.004	1.13 (1.01‐1.25)	.03
Treatment				
ChemoRT	Ref		Ref	
Induction + ChemoRT	1.05 (0.91‐1.22)	.5	NS	

**Figure 2:**
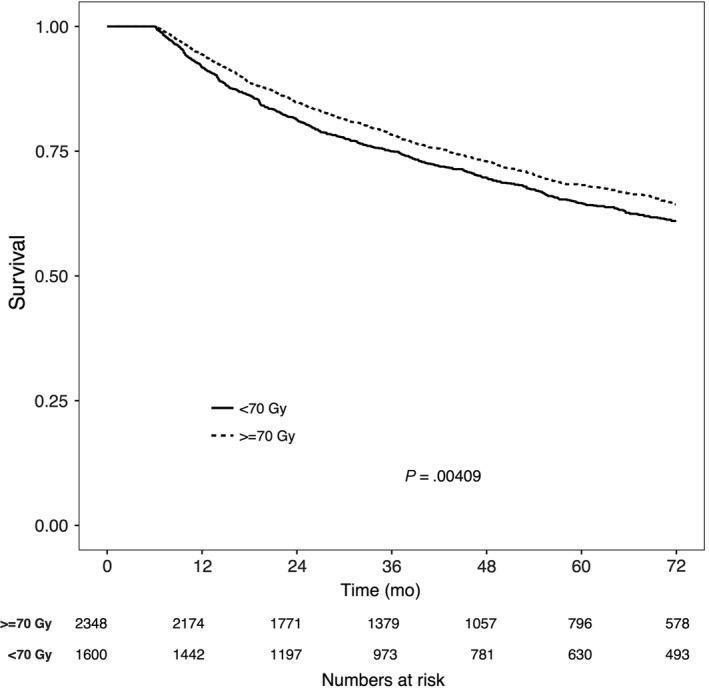
Overall survival in patients with nasopharyngeal carcinoma receiving either ≥70 Gy or <70 Gy of radiation therapy

At the time of analysis, 1566 patients (32.2%) had died. The median follow‐up time was 45 (6.0‐154.7) months. On univariable analysis, factors associated with improved OS were younger age, lower Charlson‐Deyo Comorbidity Index (CDCI), “Other” race, patients with private insurance, higher education and income level, lower T‐ and N‐stage, and patients receiving ≥70 Gy RT (Table 3). On multivariable analysis, young age, lower CDCI, “Other” race, private insurance, lower T‐ and N‐stage, and RT dose ≥70 Gy remained independent predictors of OS (Table 3). The 5‐year OS in patients receiving ≥70 Gy was 69.6% vs 67.2% in patients receiving <70 Gy (Figure 2, *P* < .01).

**Figure 3:**
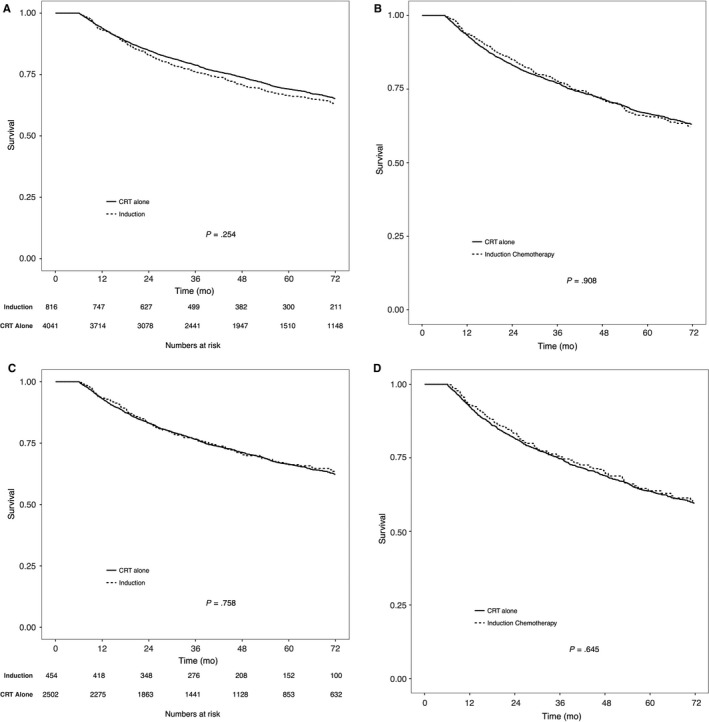
Overall survival in patients with nasopharyngeal carcinoma receiving induction chemotherapy or concurrent chemoradiation. A, Represents the whole cohort, unmatched. B, Represents the whole cohort, matched. C, Represents high risk patients defined as T3‐4N1 or TanyN2‐3, unmatched. D, Represents high risk patients defined as T3‐4N1 or TanyN2‐3, matched

Induction chemotherapy was not found to be associated with survival on either univariable or multivariable analysis. The 5‐year OS in patients who received induction chemotherapy was 66.3% vs 69.1% in patients who received concurrent CRT alone (Figure 3A, *P* = .25). To minimize the selection bias towards the use of induction chemotherapy, as well as to minimize the differences in patient characteristics between the two groups, patients in the induction group were matched to patients in the concurrent CRT alone group using propensity score matching. In the matched cohort, the 5‐year OS was not statistically different between the two groups (Figure 3B, *P* = .91). A subset analysis in patients with high risk disease, defined as T3‐T4N1 or TanyN2‐3, was also conducted (Figure 3C). There were 2502 patients in the CRT alone group and 454 in the induction group. No differences in 5‐year OS was noted between the patients who received induction chemotherapy vs concurrent CRT alone in this high risk cohort (Figure 3C, *P* = .76). Patients in the high risk cohort were also matched using propensity score matching, and no difference in OS was noted between the two groups (Figure 3D, *P* = .64). When stratifying by individual T‐stage and N‐stage, induction therapy was not found to be associated with improved OS (Figure S3). Additionally, a subset analysis was performed on cases where detailed histological information was available (WHO Type 1 vs WHO Type 2/3). A total of 1255 cases were identified of which 260 (20.7%) were WHO I and 995 (79.3%) were WHO II/III. Induction therapy was not found to be associated with improved OS with either types of histology (Figure S4).

## 4 DISCUSSION

The results of this large cohort study show that the use of induction therapy in NPC has remained stable between 2004 and 2014. When compared with concurrent CRT alone, induction chemotherapy is not associated with improved OS in patients with NPC in both unmatched cohort and matched cohort. Patients with more advanced disease (T4 and N3) and patients with lower education had a higher likelihood of receiving induction chemotherapy. Additionally, patients receiving induction therapy also were more likely to be treated with <70 Gy. On multivariable analysis, patients with higher T‐stage, higher N‐stage, higher comorbidities, and patients treated with <70 Gy were all predictive of lower OS.

The effect of adding induction therapy in NPC is controversial. However, without a major randomized study providing physicians with any sort of direction in regards to induction therapy, the use of induction therapy has remained constant over the last decade as seen in this study. Based on the patterns of care analysis, younger patients and patients with T4 and N3 disease were more likely to receive induction therapy. The use of induction therapy in younger patient is likely due to the fact that they are better able to tolerate the additional cycles of chemotherapy compared to older adults. The use of induction therapy in T4 and N3 is likely to reduce the target volumes for the definitive chemoradiation therapy. Studies have shown that use of induction therapy successfully reduces the target volumes of RT in order to avoid overdosing of critical neurological structures and reduce RT related toxicities.^16^, ^17^ For that reason, it is not surprising that we see higher use of induction therapy in these patients with very advanced disease.

A recent meta‐analysis based on individual patient data showed a survival benefit associated with concurrent CRT, however, it failed to show a survival benefit of induction chemotherapy.^5^, ^11^ Here, we show that induction chemotherapy is not an independent predictor of OS. A phase II trial of induction cisplatin, epirubicin, and paclitaxel followed by concurrent CRT vs concurrent CRT alone did not significantly improved OS or progression free survival.^18^ Similarly, a randomized phase II/III trial evaluating induction gemcitabine, carboplatin, and paclitaxel followed by concurrent CRT vs concurrent CRT alone showed no significant improvement in OS or distant failure‐free survival with the addition on induction regimen.^19^ However, these findings have not been corroborated by several other trials. A recent randomized phase III study comparing induction docetaxel, cisplatin, and fluorouracil (TPF) followed by concurrent CRT vs concurrent CRT alone showed an improved 3‐year OS with a HR of 0.54 (95% CI 0.36‐0.95, *P* = .02).^8^ Similarly, a randomized phase II trial comparing induction docetaxel and cisplatin followed by concurrent CRT vs concurrent CRT alone showed a significantly increased 3‐year OS (HR 0.24; 95% CI 0.08‐0.73, *P* = .01), as well as a trend towards improved progression‐free survival and distant control.^20^

It has been postulated that some induction studies have largely been negative due to the use of ineffective induction regimen, high treatment‐related death, and poor patient selection. For example, induction chemotherapy may only be beneficial in patients with high‐risk disease, such as T3‐4 or N2‐3. The authors of the trials with positive results with induction therapy state that it has been the use of more effective induction regimen, such as TPF chemotherapy, as well as inclusion of only patients with high risk disease that has resulted in a significant OS benefit with the use of induction chemotherapy.^8^, ^21^, ^22^, ^23^ We performed a subset analysis on patients with high risk disease and found no significant survival advantage with the use of induction chemotherapy even in the most advanced stage NPC (ie T3‐4N1 or TanyN2‐3). Additionally, when stratified by individual T‐ and N‐stage, we also did not find an improved survival even for the most aggressive NPC (ie T4 or N3). On multivariable analysis, we did see that T4 and N3 tumors were more likely to receive induction therapy, though no survival difference was noted when stratifying by these stages. Another important aspect of whether induction chemotherapy is effective or not, is the individual components that make up the induction regimen. For example, several studies have shown that TPF compared to PF is more effective regimen in the induction setting.^21^, ^22^, ^24^ However, due to the limitations of the database that was used in this study, we are unable to draw any conclusions regarding the use of various chemotherapy regimens and its effect on OS. Several randomized trials assessing various induction chemotherapy regimens are currently underway and will assist in confirming the most optimal induction regimen.^25^ It is believed that induction therapy may have more of a role with WHO type 2/3 tumors compared to WHO type 1.^26^, ^27^ However, in the present study, no differences in OS were observed with induction therapy in either of the two types. Though, it should be noted that only ~25% of the patients had appropriate information provided to group them as WHO type 1 vs WHO type 2/3. As such, no firm conclusions can be drawn with each histological subtype.

Our study also shows that on multivariable analysis, RT dose ≥70 Gy is associated with an improved OS. This is consistent with several prior studies which have also shown 70 Gy to be an optimal dose for NPC, resulting in improved local control and OS, especially when combined with concurrent chemotherapy.^28^, ^29^, ^30^ Interestingly, on multivariable analysis, we find that in this cohort, patients receiving induction chemotherapy were in fact less likely to receive ≥70 Gy. This may result in poor local control, which may negate the improved distant control with induction therapy thereby resulting in equivocal OS. Studies have shown that patients receiving induction therapy are more likely to experience grade 3 or 4 toxicity, especially hematological toxicity.^8^ It is, therefore, possible that these toxicities are inhibiting these patients from receiving curative dose of RT. As high as 8% treatment‐related deaths have been observed in patients receiving induction therapy.^24^ Similarly, patients receiving induction therapy are also less likely to complete the full dose of concurrent cisplatin with RT.^23^ It has been shown that total dose of cisplatin administered with concurrent RT is associated with local control and OS.^31^ In the a recent phase III randomized trial of induction TPF and concurrent CRT alone, it was noted that only 30% in the induction group vs 56% in the concurrent CRT completed all three cycles of concurrent cisplatin.^8^ The NCDB does not report on the number of cycles of chemotherapy administered, however, based on prior randomized studies, it is possible that the patients in the induction group did not complete all three cycles of concurrent cisplatin. These findings are also supported in induction studies DECIDE and PARADIGM for other head and neck sites where the induction arm had significantly more adverse events compared to the concurrent CRT alone arm. These studies also did not find an OS benefit with induction therapy.^32^, ^33^

The findings of this study need to be interpreted with the understanding of the limitations that are associated with the use of NCDB data. NDCB analyses are retrospective in nature and may be confounded by selection bias. In addition, the findings of the study should be considered as associations, and not interpreted as cause and effect. We performed multivariable analysis and IPTW‐adjusted analysis to minimize the selection bias, however, additional unaccounted biases may still persist. Furthermore, plasma Epstein‐Barr virus DNA load, a known prognostic marker,^34^ is not a coded variable in the NCDB and missing from our analyses. Local control and treatment related toxicities are also unavailable in the NCDB. Additionally, the use of adjuvant chemotherapy (after concurrent CRT) was not documented in the database, and for that reason, unaccounted for in the multivariable analysis. And lastly, the induction chemotherapy regimens used in this cohort are likely very heterogeneous based on varying treatment practices around the United States. NCDB does not identify the individual components of induction chemotherapy utilized, which is a major limitation of this study. Nevertheless, this study sheds important information on the debate of induction chemotherapy for NPC in a large national cohort.

In conclusion, this study suggest that compared to concurrent CRT alone, induction chemotherapy followed by concurrent CRT is not associated with improved OS in NPC. This finding also holds true even in advanced NPC such as T3‐4N1 or N2‐3 disease. Additionally, patients receiving induction chemotherapy are more likely to be treated with lower doses of RT potentially resulting in poor local control, which may be negating the beneficial effects of improved distant control with induction chemotherapy. Future clinical trials currently underway are anticipated to answer many questions that still remain with this disease.

## CONFLICT OF INTEREST

None declared.
