# Supplementary material for: Induction chemotherapy in the treatment of nasopharyngeal carcinoma: Clinical outcomes and patterns of care
Source: Cancer Med. 2018 Jul 14;7(8):3592–603. doi: 10.1002/cam4.1626 (PMC6089177; doi:10.1002/cam4.1626)

**
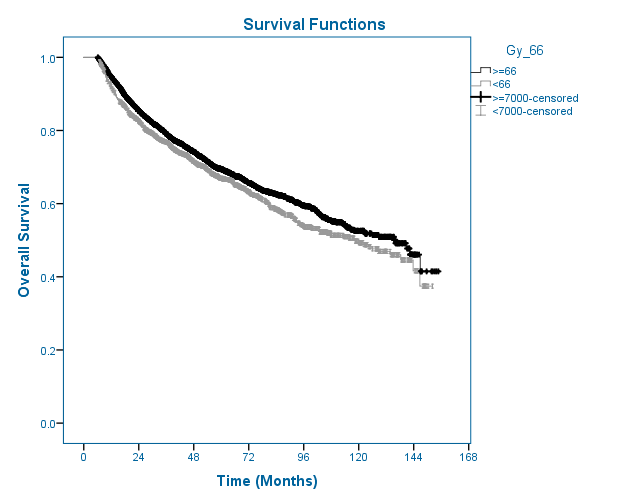

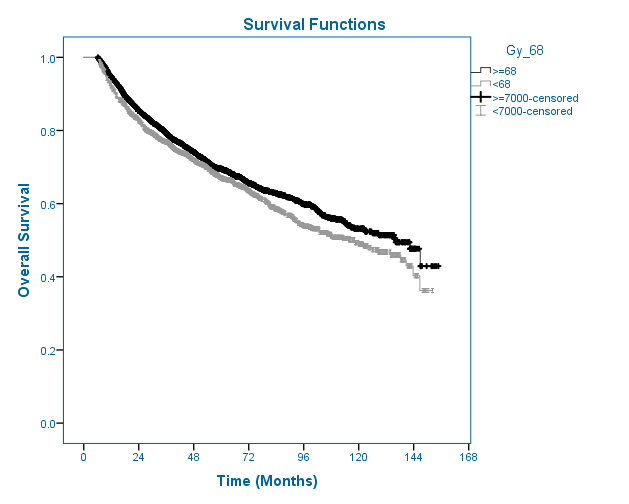
Supplemental Figure 1**. Overall Survival at each RT level from 66 Gy and 69 Gy

P = 0.004

P = 0.009

P = 0.004


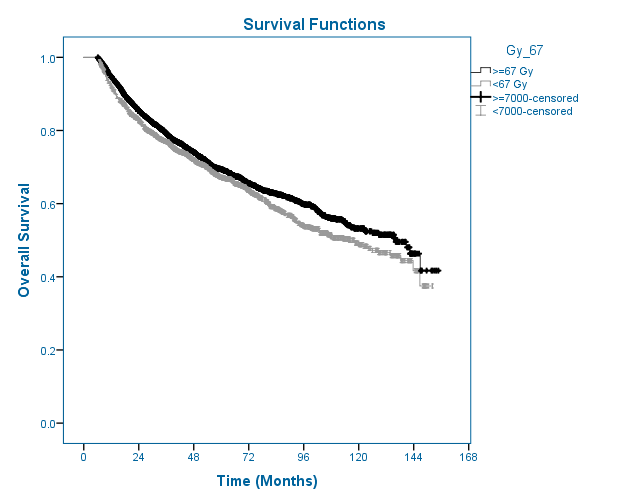

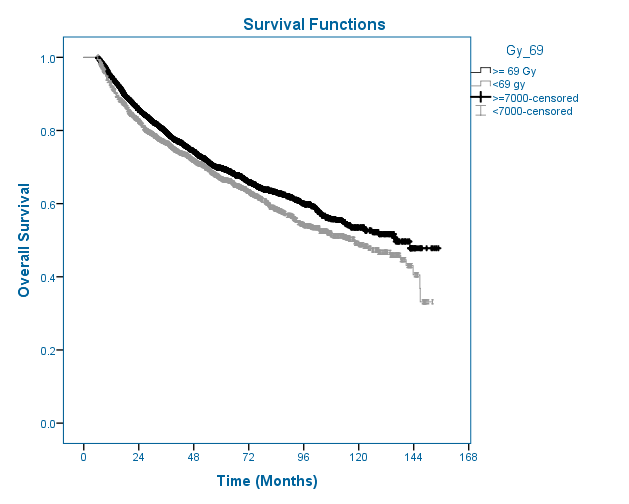


P = 0.01

P = 0.01

**Supplemental Data** **2**. Chi-Square Analyses of Induction Therapy and RT Dose for each RT level between 69 Gy and 66 Gy

Percent (%) receiving ≥ 69 Gy: 62.6% vs. 57.5% (CRT alone vs. Induction, p = 0.006)
Percent (%) receiving ≥ 68 Gy: 64.9% vs. 60.7% (CRT alone vs. Induction, p = 0.02)
Percent (%) receiving ≥ 67 Gy: 65.7% vs. 60.9% (CRT alone vs. Induction, p = 0.009)
Percent (%) receiving ≥ 66 Gy: 68.7% vs. 65.7% (CRT alone vs. Induction, p = 0.09)

**Supplemental Figure 2A. Dot-plot of standardized mean difference before (blue) and after (yellow) IPTW match for the whole cohort**


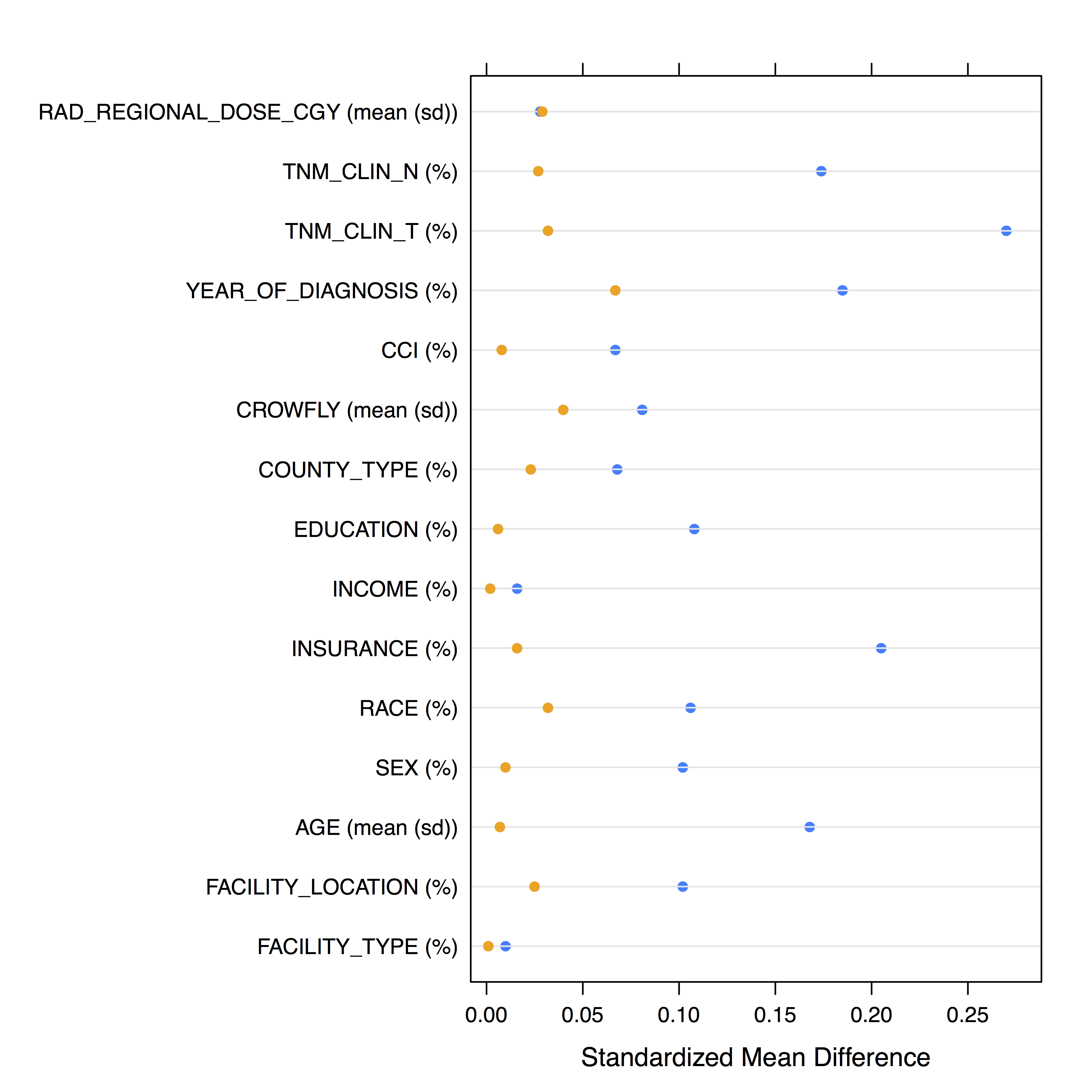


**Supplemental Figure 2B. Dot-plot of standardized mean difference before (blue) and after (yellow) IPTW match for patients with high-risk disease defined as T3-4N1 or TanyN2-3.**


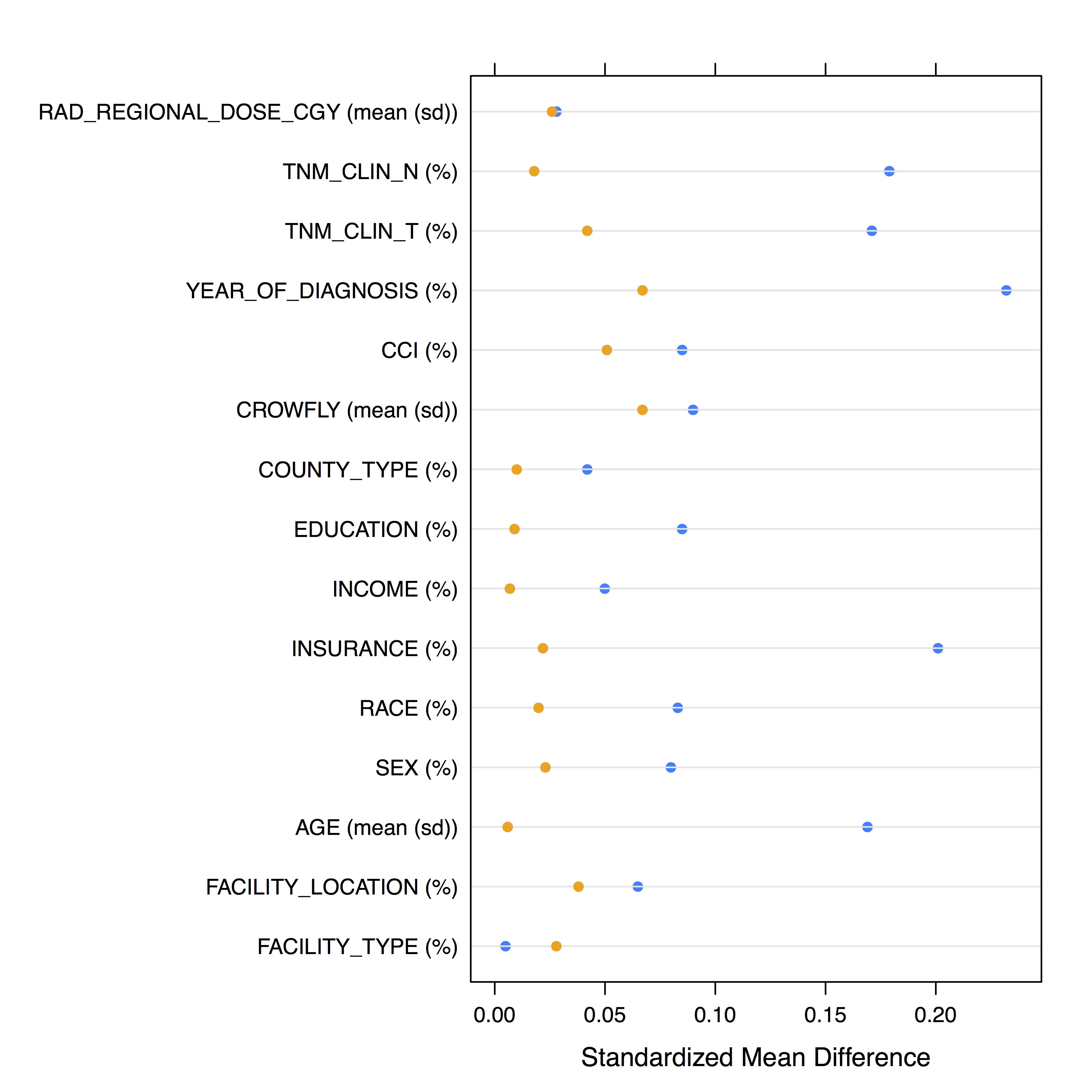


**Supplemental Figure 2C. Kernel Density Plot before and after IPTW match for the whole cohort**


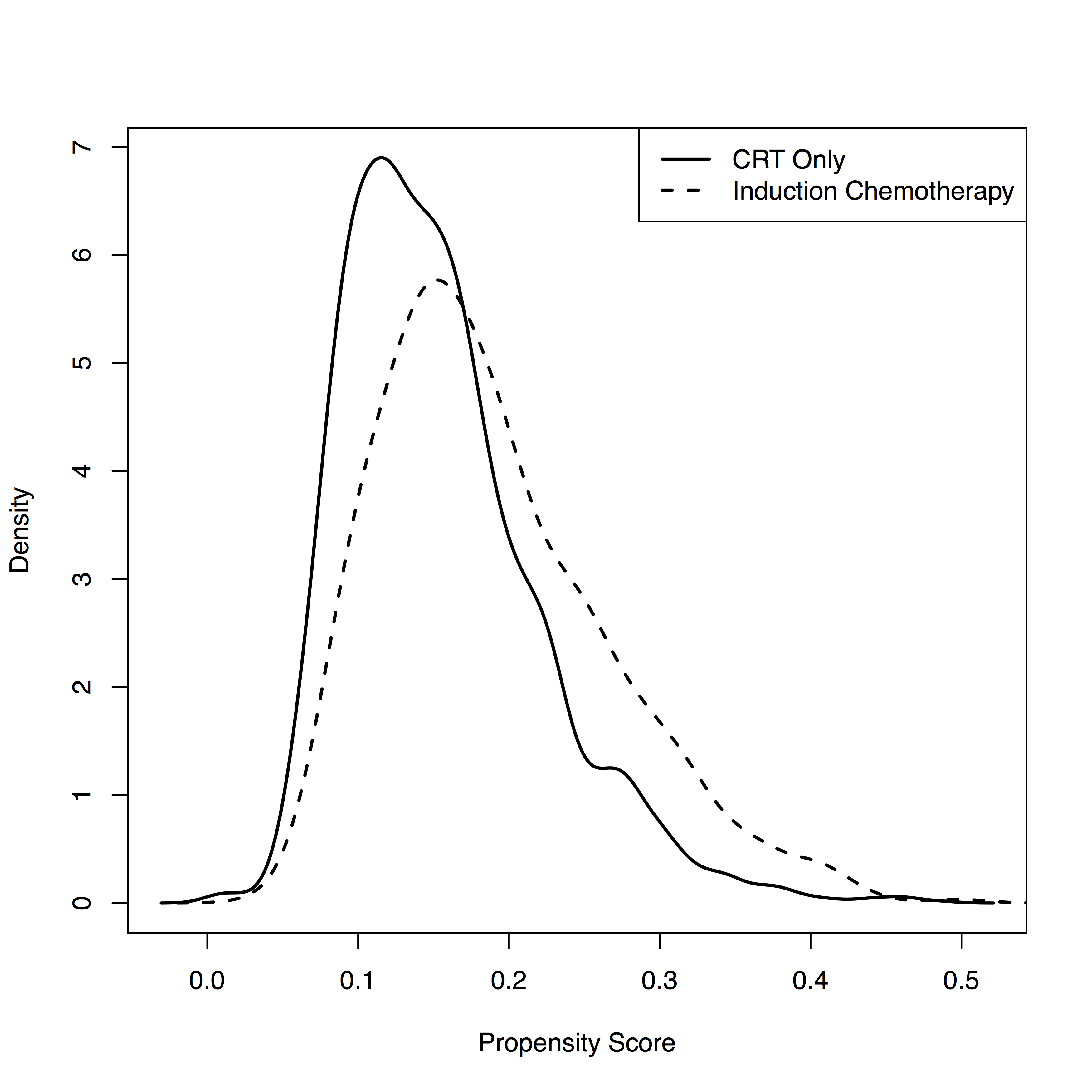

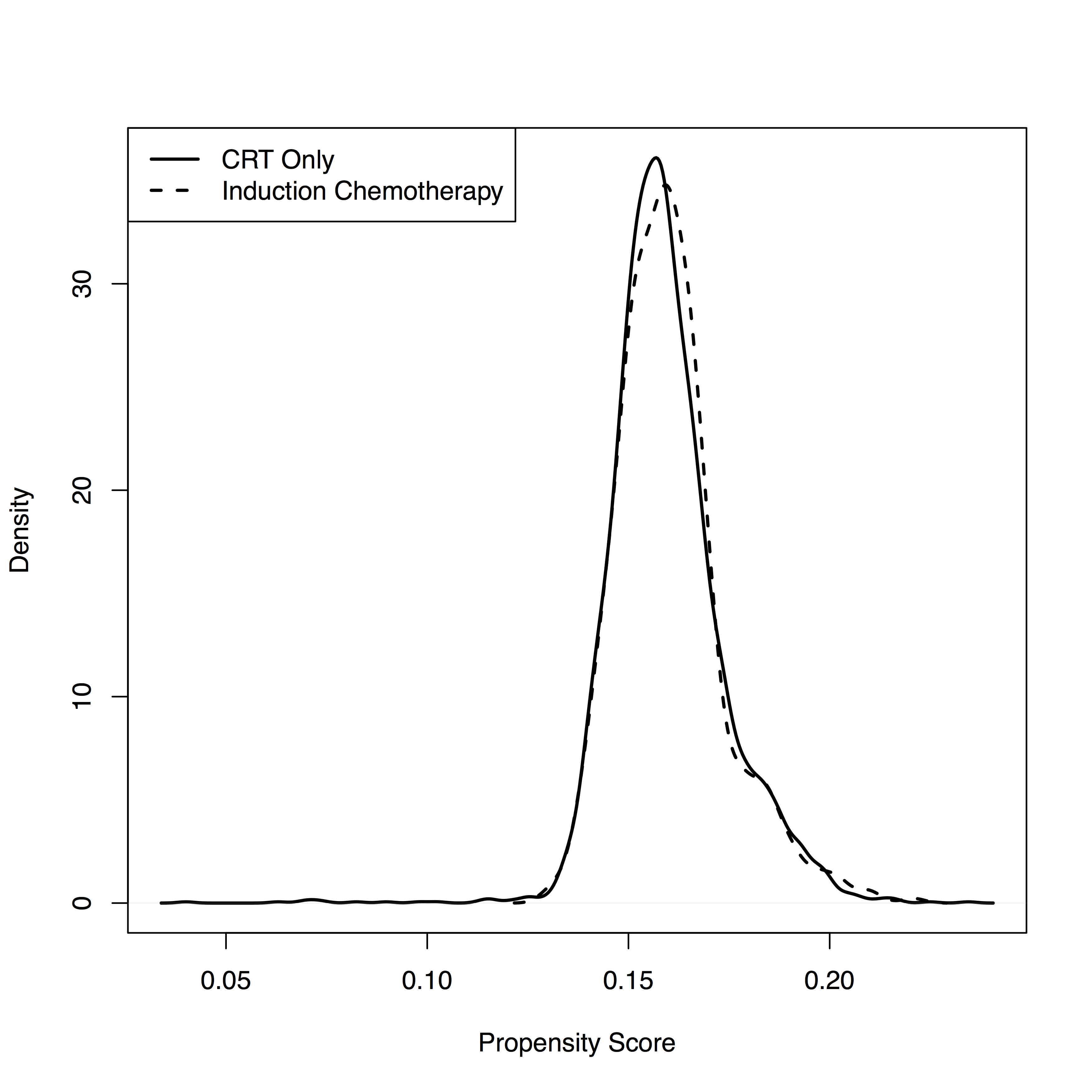


**Supplemental Figure 2D. Kernel Density Plot before and after IPTW match for the high-risk cohort (T3-4N1, or TanyN2-3).**


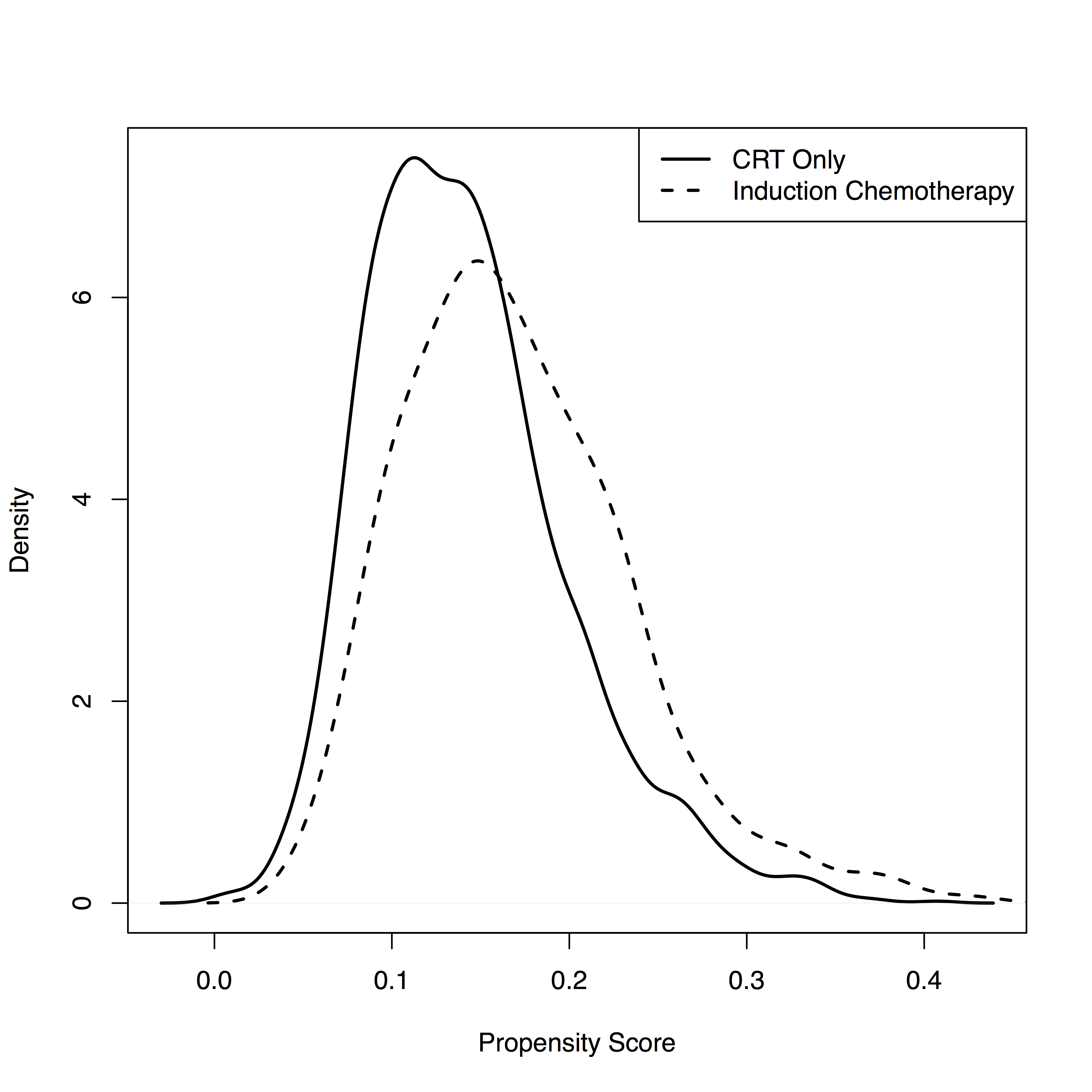

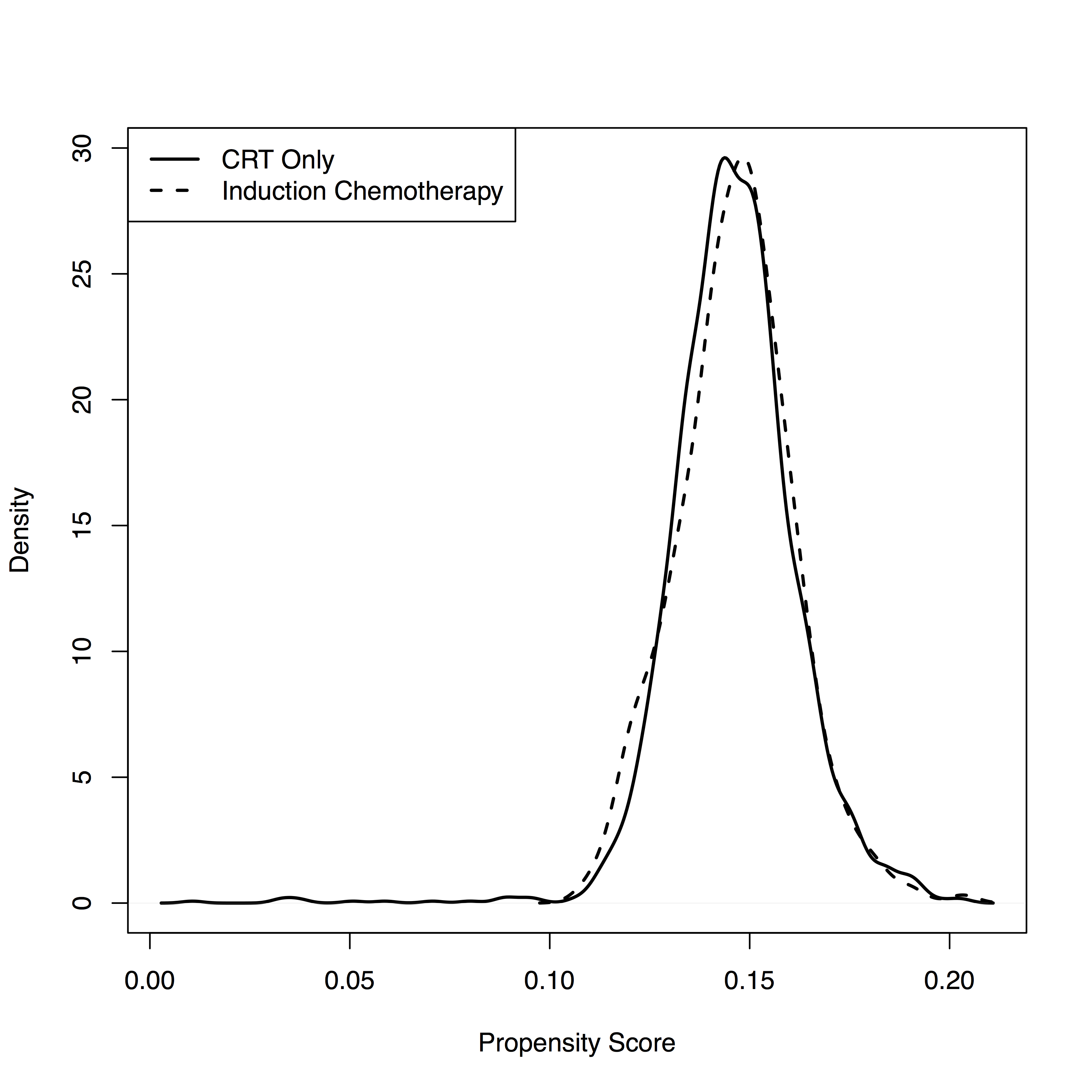


**Supplemental Figure 3.** Comparison of OS between induction chemotherapy and concurrent CRT stratified by individual T-stage and N-stage**.** There is no difference in OS at any stage.

**Stage: T1**

**
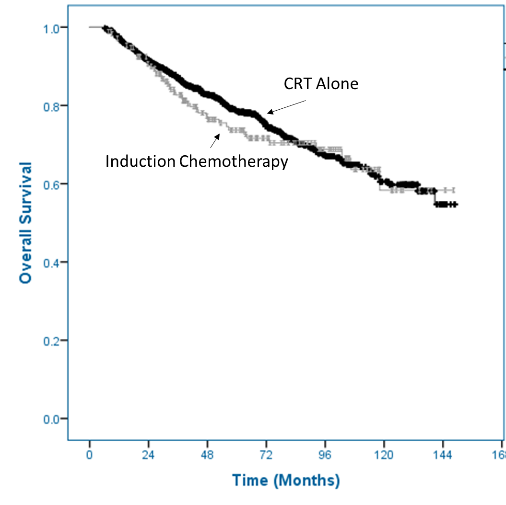
**

**Stage: T2**


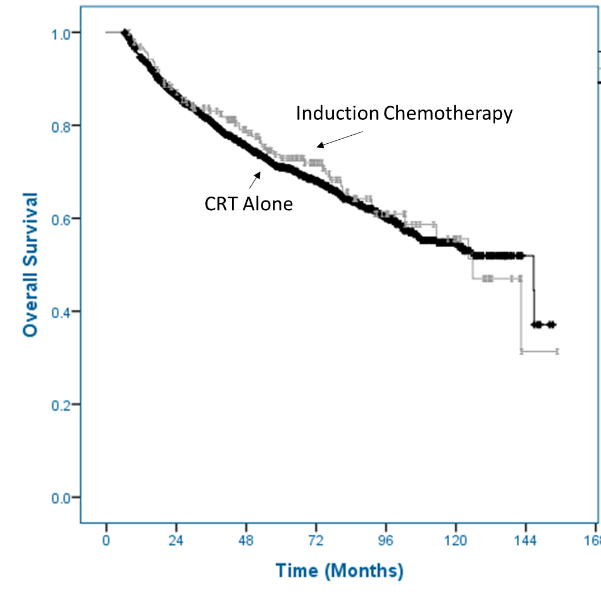


**Stage: T3**


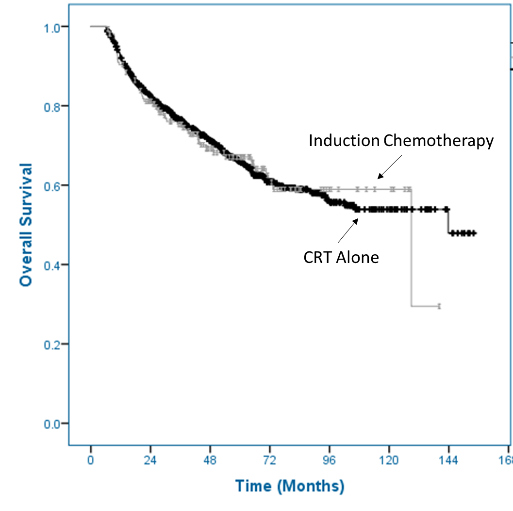


**Stage: T4**


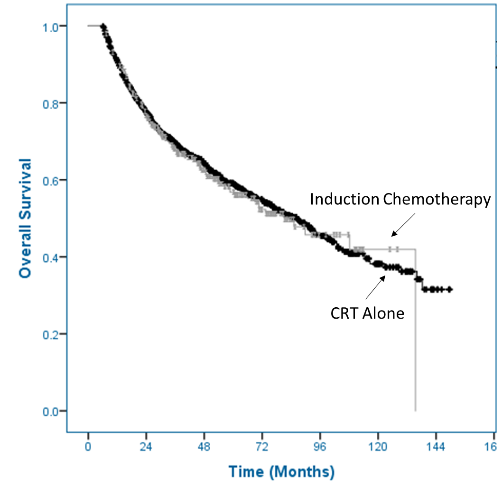


**Stage: N0**


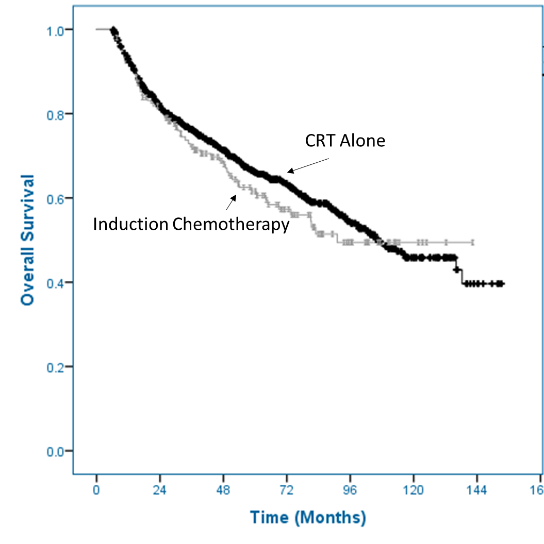


**Stage: N1**


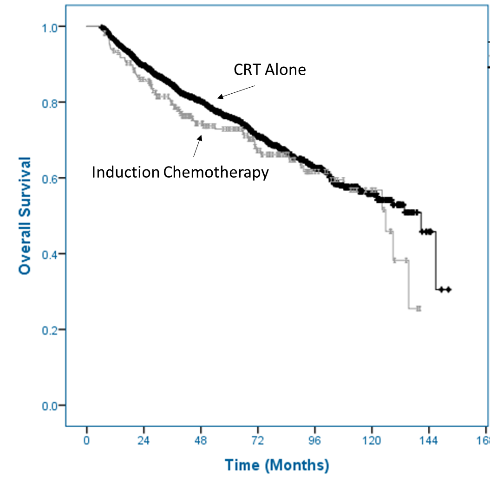


**Stage: N2**


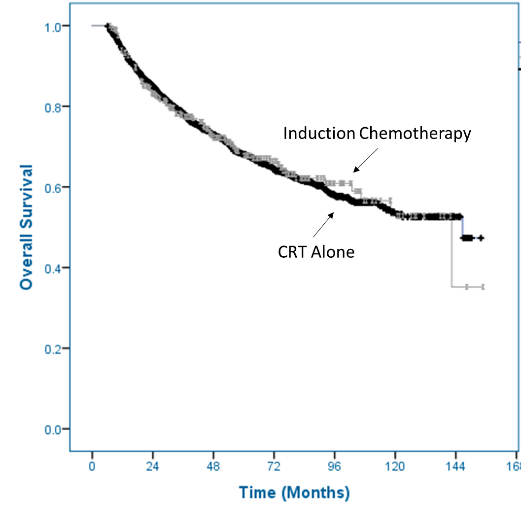


**Stage: N3**


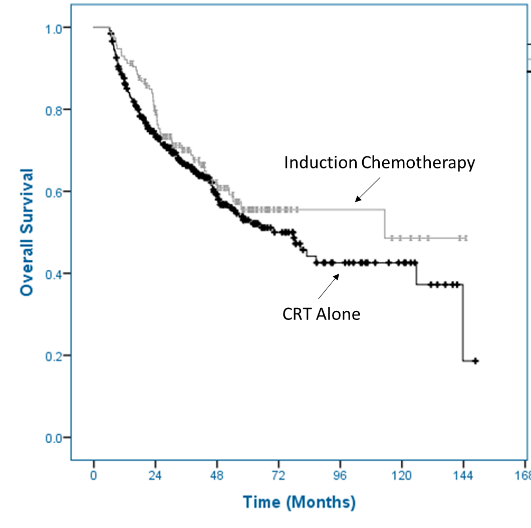


**Supplemental Figure 4.** Comparison of OS between induction chemotherapy and concurrent CRT stratified by WHO Type 1 and WHO Type 2/3**.** There is no difference in OS at for either histologies.


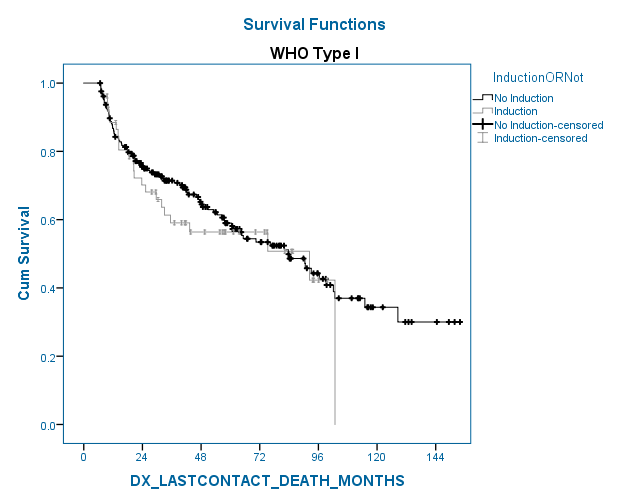


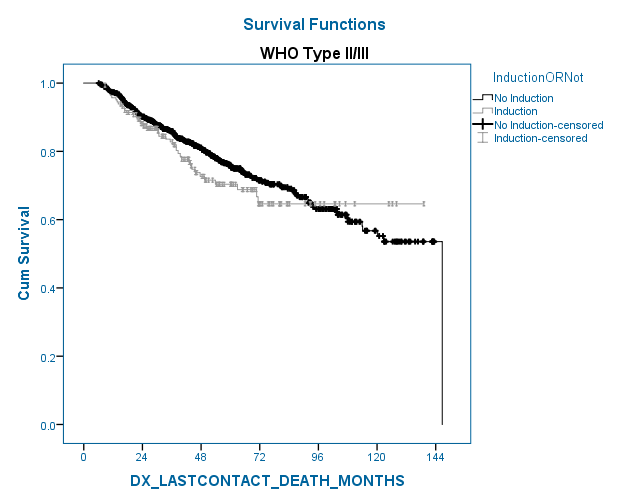

Supplement: Supplementary file 1 [file CAM4-7-3592-s001.docx]
